# Navigating the Development of Dry Powder for Inhalation: A CDMO Perspective

**DOI:** 10.3390/ph18030434

**Published:** 2025-03-19

**Authors:** Beatriz Noriega-Fernandes, Mariam Ibrahim, Rui Cruz, Philip J. Kuehl, Kimberly B. Shepard

**Affiliations:** 1 Small Molecules Product Development, Lonza Group AG, Bend, OR 97701, USA; beatriz.fernandes@lonza.com (B.N.-F.); mariam.ibrahim@lonza.com (M.I.); ruialberto.teixeiracruz@lonza.com (R.C.); 2 Lovelace Biomedical Research Institute, Albuquerque, NM 87108, USA; pkuehl@lovelacebiomedical.org

**Keywords:** dry powder inhalers, respiratory delivery, CDMO, spray drying, jet milling, capsule filling, pre-clinical studies

## Abstract

Interest in pulmonary/nasal routes for local delivery has significantly increased over the last decade owing to challenges faced in the delivery of molecules with poor solubility, systemic side effects, or new modalities such as biologics. This increasing interest has attracted new stakeholders to the field who have yet to explore inhaled drug product development. Contract development and manufacturing organizations (CDMOs) play a key role in supporting the development of drug products for inhalation, from early feasibility to post marketing. However, a critical gap exists for these newcomers: a clear, integrated, and a CDMO-centric roadmap for navigating the complexities of pulmonary/nasal drug product development. The purpose of this publication is to highlight the key aspects considered in the product development of inhaled dry powder products from a CDMO perspective, providing a novel and stepwise development strategy. A roadmap for the development of inhalable drug products is proposed with authors’ recommendations to facilitate the decision-making process, starting from the definition of the desired target product profile followed by dose selection in preclinical studies. The importance of understanding the nature of the API, whether a small molecule or a biologic, will be highlighted. Additionally, technical guidance on the choice of formulation (dry powder/liquid) will be provided with special focus on dry powders. Selection criteria for the particle engineering technology, mainly jet milling and spray drying, will also be discussed, including the advantages and limitations of such technologies, based on the authors’ industry expertise. Lastly, the paper will highlight the challenges and considerations for encapsulating both spray dried and jet milled powders. Unlike existing literature, this paper offers a unified framework that bridges preclinical, formulation, manufacturing, and encapsulation considerations, providing a practical tool for newcomers.

## 1. Introduction

Pulmonary drug delivery remains the preferred route of administration for treating lung diseases. Moreover, the pulmonary route offers rapid absorption and minimal enzymatic activity compared to oral delivery, making it a competitive route for systemic delivery [1,2]. Similarly, nasal delivery is gaining momentum as it offers mucosal immunity, central nervous system delivery, and local and systemic delivery opportunities [3]. Treatment of local diseases coupled with poor oral bioavailability and/or concerning systemic side effects are notably the most common drivers for innovators to consider the inhalation route of delivery. Improved patient compliance, portability, and stability at room temperature are additional major gains that could potentially provide a differentiated product compared to injectables [4].

The innovators, especially those with limited/no prior expertise regarding the unique considerations of the inhalation route, face the hurdles of deciding on the dose, formulation, process, and device for optimum drug delivery to achieve the target product profile (TPP) desired for each indication. There is a wealth of knowledge about pulmonary/nasal delivery from the abundant literature in this field [5,6,7,8,9,10]. However, there is no clear and stepwise guidance on selection of the dosage form, formulation, and the manufacturing process while considering the nature of the API, the dose range, and the drug product development phase. Additionally, the integration of different fields of expertise, including formulation, analytical sciences, pharmaceutical engineering, and device design, etc., needed for a successful product development, is often limited to the industry sector. Contract development and manufacturing organizations (CDMOs) are companies that provide technical expertise, manufacturing capacity, and/or regulatory guidance, supporting the innovators with their drug product development needs [11]. CDMOs with expertise in the inhalation field support the development of inhaled products, from early feasibility to post marketing depending on their capabilities. This paper will provide research and experienced-based opinions on the best approaches to tackle the unknowns related to inhaled product development for pulmonary and nasal delivery from a CDMO perspective, from preclinical development through encapsulation of dry powders. Aspects related to device design, selection, and testing are not covered in this paper.

## 2. The Target Product Profile Defines the Road Map for Product Development

The definition of the desired TPP is the first step towards the development of a drug product. According to the World Health Organization (WHO), TPP is the desired ‘profile’ or characteristics of a target product that is aimed at a particular disease or diseases. TPPs state intended use, target populations, and other desired attributes of the products, including safety and efficacy-related characteristics [12]. Other attributes in the TPP include therapeutic dose range, dosing frequency, and intended storage conditions.

The TPP serves as a guidance for development teams to decide on the delivery approach, the manufacturing technology, and consequently the formulation. It is important to note that TPP must be realistic, achievable within the planned timeframe, phase appropriate, and considerate of the physicochemical properties of the API [3]. For instance, it is not feasible to formulate an API with large therapeutic dose as a dry powder inhaler (DPI) if it exceeds the maximum tolerable amount of inhalable dry powder per dose (approximately 100 mg/capsule) [13]. As a second example, an inhalable vaccine product intended for room temperature storage should be formulated as dry powder to maintain its stability for a reasonable shelf-life duration [14].

The following sections will explore the interplay between the TPP, the dose, API characteristics, formulation selection, specifically addressing liquid versus solid formulations, particle engineering technologies, and encapsulation process considerations.

## 3. Pre-Clinical Development: Define Your Dose

Key parameters in defining the TPP are the therapeutic dose, the No Observed Adverse Effect Level (NOAEL), and the No Observed Effect Level (NOEL) doses. Hastedt et al. emphasized the crucial role of understanding the target dose in selecting the appropriate formulation and delivery device [15]. This is evident when examining marketed products within each formulation/device category.

The project team is responsible for developing the TPP, with the dose often serving as a central parameter. Typically, the therapeutic dose is initially defined in non-clinical studies, encompassing pharmacology, pharmacokinetic (PK), and/or toxicology studies. Subsequently, it is refined in Phase I and Phase II clinical studies. It is crucial to remember that non-clinical models are just that—models. The team should not expect the dose determined in preclinical studies to translate directly to the clinical setting.

Early in drug discovery, when focusing on proof-of-concept pharmacology and pharmacokinetic studies, the primary objective is often the detection of a positive signal rather than the dose or the dose response curve. As such, simple aqueous or suspension formulations delivered with simplistic devices, including laboratory pipettes, can suffice. As the pharmacology models move from signal detection to dose-response relationship and target tissue engagement, both the formulations and delivery technologies become more sophisticated. For an inhalation program, this typically involves moving from a bolus delivery to the lungs (e.g., intratracheal delivery) to inhalation delivery. This initial inhalation delivery may continue to employ simple formulations but delivered with approved devices such as nebulizers [16].

At this stage of development, there is a greater focus on dose calculation, dose consistency, and the translatability of the dose to clinical models. It is common for a dose to change by a factor of 2 to 10-fold (typically a reduction in dose based on body weight) when transitioning from a bolus delivery to an inhalation delivery [17]. The dose delivered by inhalation (regardless of formulation or delivery method) should be calculated as defined by Tepper et al., accounting for all potential losses encountered during aerosolization, administration, loss during absorption and metabolism, and, finally, loss of drug during distribution and delivery to the intended target site, particularly relevant for systemic delivery [7].

Relying on inhalation delivery in a pharmacology/pharmacokinetic study provides the team with sufficient confidence to include the dose in the TPP decision making process. The TPP will be based on dose, solubility, physico-chemical properties, patient population, etc. The selected formulation that meets the TPP can then be utilized in additional pharmacology/pharmacokinetic studies (not required but may be scientifically justified) and regulatory toxicology programs. Importantly, the regulatory toxicology studies should ideally include a formulation and device that closely resemble the intended clinical approach.

Throughout product development, innovators often have questions regarding the optimum timing for locking the final formulation and the risk(s) associated with formulation changes post preclinical studies. There is no single answer to these questions. Instead, the scientific development team should consider the extent of any formulation changes, their impact on the TPP, the team’s risk tolerance, and the overall project development plan to determine the necessary level of additional work or studies.

## 4. Understand Your API: Small Molecules Versus Biologics

The API’s nature, whether it is a small molecule or a biologic, significantly influences CMC decisions and the product development path. CDMOs offering inhalation product development services are expanding their capabilities to handle both small and large molecules to meet the current diverse market demand.

The key physicochemical properties of small molecule APIs include solubility, crystallinity, polymorphism, thermal stability, and any known chemical susceptibility. Solubility is crucial as it dictates the suitability of solution-based inhalers. Moreover, it guides the selection of particle engineering technology for the manufacture of inhalable dry powder, which will be discussed later. Evaluation of the pulmonary solubility and permeability of the selected molecule could provide a valuable insight regarding the anticipated dissolution and absorption profiles of the API post-inhalation. The inhalation-based biopharmaceutics classification system (iBCS) classifies inhaled drugs based on their pulmonary permeability and solubility for the target lung dose, providing a framework for predicting their dissolution and absorption behavior in the lungs. Class I drugs exhibit complete dissolution, rapid absorption, and clearance for the target dose; Class II, incomplete dissolution and dissolution-dependent clearance; Class III, complete dissolution and permeability-dependent clearance; and Class IV, incomplete dissolution and permeability-dependent clearance [18]. This classification should guide the formulation strategy to meet the desired TPP and reduce risks of unexpected PK profiles. For instance, formulating an API in its amorphous form with a glass former like trehalose may enhance dissolution rates compared to crystalline micronized forms, which can lead to delayed release rates. For a dissolution-limited molecule such as fluticasone propionate (iBCs II), extended lung exposure is anticipated, but its dissolution rate has been improved through formulation within an amorphous matrix [19]. Conversely, systemic delivery of iBCS III or IV molecules (low permeability) requires alternative formulation strategies, such as incorporating surfactants or other permeability enhancers [20]. Alternatively, the dose and the dose regimen could be adjusted accordingly to accommodate the limitations of the iBCS class of the drug, if feasible.

Inhaled biologics are non-invasive and more convenient for patients than injectables. Biologic APIs, such as proteins, peptides, and nucleic acids, are complex, high molecular weight molecules with distinct physicochemical properties depending on their modality. Biologic molecules are susceptible to a variety of stresses that could negatively impact their integrity, leading to loss of efficacy and/or potential immunogenicity concerns [21]. Thus, the development of a biologic product for pulmonary or nasal delivery requires a distinctive set of analytical capabilities and expertise to support the characterization of the biologic molecule as well as the aerosol product [22]. Characterization of biologics typically involves assessing molecular weight, structure, purity, aggregation propensity, charge, melting temperature, and solubility [23].

In terms of formulation, the selected dosage form, whether it is liquid or dry powder for inhalation, will impact the excipient selection in the formulation. Buffer salts, sugars, lipids, polymers, and surfactants are typically used in both liquid solutions and dry powder biologics to maintain their integrity [24,25]. It is noticeable that early stages of preclinical and clinical studies are mostly conducted using liquid biologics solutions for inhalation [26]. The preference for liquid formulations of biologics during such “proof of concept” stages could be attributed to the simpler formulation and the easier manufacturing process of liquid solutions compared to dry powders [27]. In addition, the wealth of expertise in the formulation of biologics as liquid solutions for injections may potentially be the driver to first explore liquid solution formulations. However, the advantages offered by dry powders for inhalation make the transition from liquid to dry powders appealing. The optimal strategy—whether to pursue both formulations in parallel, begin with dry powder, or switch after Phase I—is a decision best made on a project-by-project basis, and is explored in the following section.

It is important to highlight that while regulatory guidance for inhalation products is well defined, there is a lack of clarity regarding the expectations of inhaled biologics regulations compared to small molecules ones [28]. For instance, there is no clear guidance on the acceptable limits of protein aggregates, which may introduce immunogenicity risks [21]. Innovators of inhaled biologics rely on established regulatory knowledge related to small molecules or parenteral formulations of biologics, both of which are not sufficiently relatable to inhaled biologics. Consequently, the scientific community for inhaled medicine is actively discussing the manufacturing challenges and the regulatory gaps for pulmonary or nasal biologic products. CDMOs are participating in scientific forums to voice the need for a clear regulatory guidance for inhaled biologics in order to be fully equipped to assist their clients accordingly.

## 5. DPI Is Recommended When Feasible

To effectively deliver drugs to the lungs, several delivery technologies are employed: pressurized metered dose inhalers (pMDIs), nebulization, soft mist inhalers (SMIs), and DPIs [28].

Historically, pMDIs have been employed mainly for the management of asthma. pMDIs use a pressurized propellant to generate the aerosol with a measured dose of medication. While pMDIs benefit from a well-established technology, they also present limitations, including the necessity for patient coordination to ensure effective drug delivery, limited applicability for diverse API modalities, and a significant environmental impact due to propellants [29]. Consequently, interest in pMDIs has diminished.

Nebulizers offer an alternative by converting liquid medication into a fine mist, independent of the patient’s inhalation technique, allowing for large delivery doses. This makes them particularly suitable for infants, young children, the elderly, and individuals with breathing difficulties. However, nebulizers require a power source and can be time-consuming to use.

SMIs provide another option, offering portability and the ability to deliver larger doses with less reliance on precise coordination between actuation and inhalation compared to pMDIs. This is achieved by extending the duration of the aerosol cloud. SMI limitations include the requirement of careful formulation development due to the impact of factors like solubility and viscosity on aerosol performance, especially with high drug concentrations in small volumes. This necessitates strategic excipient selection for poorly soluble drugs, sterility assurance, and dose uniformity management, particularly for suspensions, to prevent nozzle clogging [30]. Moreover, SMI technology currently necessitates that the medication be in liquid form, which may present challenges related to cold chain storage and transportation.

DPIs, however, deliver dry powder directly to the lungs, offering advantages such as propellant-free delivery and high efficiency without a power source. Other advantages include minimal patient-device coordination to achieve good aerosolization [31]. Additionally, DPI formulations exhibit superior chemical stability compared to liquid formulations, which is a major consideration, especially for biologic therapies that have limited liquid stability and otherwise require cold storage conditions [32]. These factors, along with historical advancements in DPI technology [33], have contributed to the growing interest in this delivery method. In fact, DPIs currently lead the market in terms of sales and revenue [11]. The global inhaler device market was worth USD 837 million in 2020 and is projected to increase the compound annual growth rate (CAGR) by 5.2% from 2022 to 2027 [12]. Therefore, the authors support selecting dry powder for inhalation as a first choice for a dosage form for pulmonary or nasal delivery if feasible.

A dry powder formulation requires key components, including aerosol enhancing excipients, diluents, and stabilizers. The selection of such excipients depends on the type of API molecule and their physicochemical properties. For instance, reducing sugars such as lactose cannot be used with proteins as they interact with amino acids through the Maillard reaction [34]. Similarly, mannitol, a crystalline diluent, is often not used with proteins as they require an amorphous matrix to limit their molecular mobility to enhance their stability [35]. Amino acids, such as leucine and trileucine, are often included to enhance the aerosol performance of powders and act as shell forming excipients, offering moisture robustness and protection for the biologic molecule in the particle core [36].

A primary question researchers must address is whether sufficient API is available for DPI formulation feasibility studies. Feasibility assessments determine if the API can be effectively delivered in solid form, addressing questions such as: Can the API be isolated in a stable crystalline form? Can the crystalline form withstand the stress of milling to inhalation-appropriate particle sizes? For spray drying, is there a suitable solvent for the API, and is the API thermosensitive? These critical questions are to be addressed early in development.

Following feasibility, additional API is needed for formulation development/optimization, process development, and scale-up to support preclinical and clinical supplies. If sufficient API is unavailable, initial clinical trials may be conducted with a simple liquid nebulized formulation. Upon successful Phase I trials and with a scaled-up API manufacturing process, transitioning from a liquid to a solid DPI formulation remains possible, provided the formulation development aligns with the TPP. However, this transition may necessitate additional toxicity and pharmacokinetic bridging studies. Thus, initiation of product development as DPI is recommended when possible if it aligns with the desired TPP.

**Figure 1:**
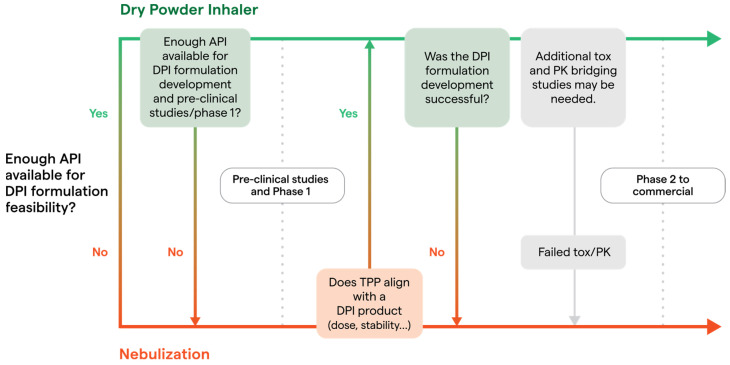
Decision Roadmap for Inhalation Product Formulation: DPI vs. Liquid.

Figure 1 offers a practical guide for navigating the development pathway for DPI products. This roadmap assists researchers with the decision-making regarding initiation of a DPI formulation from the preclinical stage or post transition from a liquid formulation during subsequent development phases. The figure also identifies key obstacles that often lead to the selection or retention of liquid-based inhalation therapies, notably limited API availability, problematic tox/PK findings, and hurdles in DPI manufacturing.

## 6. DPI Manufacturing: Spray Drying or Jet Milling

Currently, the two main established technologies for DPI manufacture are jet milling followed by blending for carrier-based formulations and spray drying. New emerging technologies, such as spray freeze drying, thin film freezing, critical spray drying, etc., offer alternative approaches for micronization; however, they are not widely implemented, single-sourced, or have limited scale up capabilities [5].

Jet milling is a widely employed, scalable technique for producing fine powders with a narrow particle size distribution. A jet mill operates on the principle of high-velocity gas streams. Particle breakage relies on high-energy collisions between the particles driven by high-pressure gas inside a milling chamber [37]. After jet milling, most formulations rely on blending of micronized API with lactose to develop carrier-based DPIs. Blending facilitates the dispersion of the micronized API on the surface of lactose to enhance its aerosolization upon inhalation. The addition of fine lactose particles, similar in size to the API, can further improve the dispersion and release of the API [38]. In some cases, force-control agents such as magnesium stearate may be incorporated into the formulation to enhance the binding of the API to the carrier (Breo^®^ ELLIPTA^®^ is an example of a commercial product containing magnesium stearate as a force control agent).

The traditional carrier-based approach to formulating DPIs is the most widely used formulation approach. The majority of marketed DPIs for high-potency respiratory medications, such as fluticasone, salmeterol, and tiotropium, have traditionally relied on micronized drug particles blended with lactose. The carrier-based formulation strategy offers many benefits. It relies on standard technologies (milling followed by blending) using commercially-precedented excipients and an API with a stable crystal structure, avoiding any phase transitions during storage, handling, or processing.

One limitation for carrier-based formulations is that optimal performance and stability are typically achieved at relatively low drug loads, often below 10–15% of the total formulation weight [39]. This limitation necessitates higher powder loads to achieve the desired therapeutic dose, potentially impacting patient convenience and device design. Additionally, carrier-based formulations are not suitable for biologic molecules due to the high energy involved during milling, which is anticipated to cause denaturation and major stability concerns. Also, jet milling is not recommended for biologic APIs supplied in a liquid form since an initial drying step is required prior to milling, further complicating the DPI manufacturing process.

Spray drying is the second most used manufacturing technology for inhalable dry powder in the pharmaceutical contract manufacturing industry. Spray drying offers a semi-continuous process for particle engineering through the atomization of API solution/suspension into fine droplets that are rapidly dried into solid particles. It offers controlled particle engineering through pre-selected formulation and process parameters to achieve the desired particle size for pulmonary or nasal delivery and accommodates significantly higher drug loads [40,41,42]. Also, spray drying is the main technology used for making inhalable dry powder for biologics. The potential degradation due to thermal or mechanical stresses during spray drying could be mitigated through careful selection of excipients in the formulation, such as surfactants, surface enriching excipients, polymers, etc. Additionally, optimizing the spray drying process variables is crucial to successfully manufacture DPI while preserving protein quality [43,44]. For instance, the outlet temperature requires adjustment to a level that guarantees sufficient drying with minimal impact on protein stability. If not feasible, secondary drying should be considered to avoid the detrimental effects of high outlet temperatures on protein stability [42].

Development of a spray drying process can be more challenging compared to jet milling and blending. Spray drying throughput relies on the API and excipients solubility in the solvent system to be sprayed, which could be limiting factors if working with a poorly soluble API, resulting in low throughput [44]. Lastly, a prominent disadvantage for spray dried products is their poor flowability properties that adds on challenges through the encapsulation process compared to carrier-based ones [45].

From a process perspective, both spray drying and jet milling can achieve high yield and high-throughput production with well-defined critical process parameters and design spaces, especially when executed by experienced teams. However, jet milling necessitates a subsequent blending step, typically a batch process with good yield. Scaling up blending, particularly for low API loads and ensuring homogeneity, presents challenges that are often API-specific. Increased API load in the blend can reduce uniformity issues but may compromise powder flowability and yield [39]. As with particle engineering, development expertise is crucial for achieving a ‘right first time’ outcome and meeting clinical timelines.

**Figure 2:**
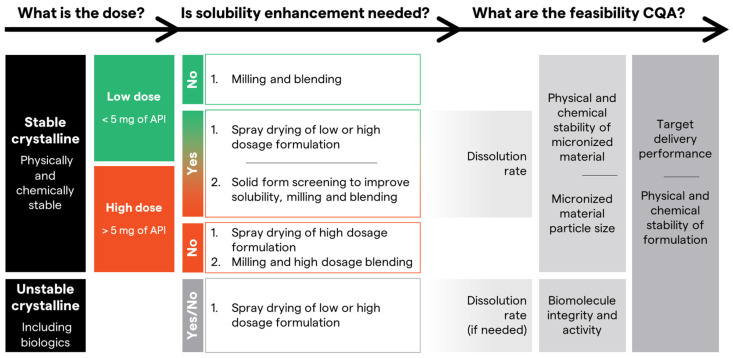
Recommendations for Formulation Screening Strategies for Low and High Dosages in Relation to API (Small Molecule vs. Biologics) and its Physicochemical Properties for Pulmonary DPIs.

Therefore, the pros and cons of jet milling and blending versus spray drying should be carefully assessed to choose the appropriate technology for the DPI, considering the TPP. The nature of API, particularly its physicochemical characteristics like crystalline stability under jet milling’s high-energy conditions, and the dose ranges are major determinants of the micronization technology as shown in Figure 2. When both technologies are feasible, other logistics and business-related considerations, such as throughput, scale up, cost, etc., should also be included in the decision process.

## 7. DPI Encapsulation: Powder Flowability Matters

DPIs are considered combination drug products that rely on powder filling in reservoir, blister, disc, or capsule-based devices [29]. Gravitational forces are not sufficient for uniform filling of a dosing receptable with inhalable dry powders due to their poor flowability and the frequent necessity of micro-dosing below 20 mg. Therefore, current filling methods require application of either mechanical deformation/compaction (dosator, screw auger) or vacuum or pressure drop (drum filler, membrane filler) forces to overcome the powder flowability limitations [46]. Encapsulating carrier-based formulations, particularly those using coarse lactose as a bulking agent, often presents fewer challenges than spray-dried formulations due to their superior flowability. However, ensuring content uniformity can be more challenging for carrier-based formulations due to possible segregation during the filling process.

The most used filling technologies for inhaled dry powders are the dosator and vacuum drum filling systems. In a dosator system, the fill-weight is easily adjusted with process parameters, controlling the powder volume [47]. It is more commonly used for powders showing Carr’s Index of approx. 15–30% (good, fair to passable), making it better suited for carrier-based formulations. However, it has inferior performance with lower doses (below 15 mg) and poorly flowing powders. In contrast, the vacuum drum filling system is very well suited for low doses and poorly flowing powders, but with a lower degree of fill-weight flexibility.

In addition to the technology’s impact on target fill-weight, the encapsulation technology’s influence on product performance must also be considered. Powder compaction during filling, whether by vacuum pressure or dosator compression, may improve the filling precision and consequently increase the encapsulation process yield. However, powder compaction can negatively impact product performance by hindering powder emission and dispersibility as it increases powder bulk density, significantly affecting flowability [48,49]. Therefore, encapsulation process development requires a balance between process optimization and product performance. CDMOs work closely with the equipment manufacturers of each filling technology to ensure successful encapsulation.

When developing an encapsulation process, it is important to characterize the formulation’s powder rheology under different environmental conditions, such as temperature and relative humidity [46], since they can inform the best filling technology and the optimum filling process conditions. It is also important for the filling process to be phase appropriate. For instance, hand filled capsules are acceptable for most pre-clinical work. The development of a viable and automated filling process becomes necessary when GMP and manufacturing volume requirements become a factor. Bridging studies may be needed as the product moves along different stages of development to compare analytical test results, including assay, dosage form uniformity, emitted dose, and aerosol performance, to ensure consistent and comparable outcomes between hand-filled, small-scale, and large-scale filling technologies.

## 8. Conclusions and Future Remarks

Inhaled drug delivery offers significant therapeutic potential, extending beyond local treatment to encompass systemic drug delivery. While these routes hold immense promise, their full potential remains largely untapped. The development of a successful inhaled product necessitates a well-defined TPP that serves as a roadmap throughout the entire development journey.

This TPP begins with pre-clinical studies to define the therapeutic dose range. While liquid formulations may be suitable for early stages of development, DPIs are often recommended when feasible due to their advantages, including long-term stability, patient convenience, and the elimination of the need for cold chain storage.

Subsequently, a deep understanding of the API’s characteristics—whether it is a small molecule or a biologic—is crucial. This understanding informs the selection of appropriate particle engineering techniques for DPIs, such as spray drying or jet milling. By meticulously considering these factors, researchers can increase the likelihood of developing safe and efficacious inhaled therapies.

The success of an inhaled product hinges upon a robust partnership between innovators and CDMOs. CDMOs play a pivotal role in this journey, bringing expertise in formulation development, manufacturing processes, and scale-up. To effectively support the growing demand for inhaled products, CDMOs need to be equipped with enhanced expertise, proactive thinking, and the readiness for scale up operations to meet the evolving needs of the field.

This research provides a comprehensive CDMO-centric framework for navigating the complexities of DPI development, enabling stakeholders to make informed decisions at each stage. By bridging the gaps from preclinical studies to final product encapsulation, this work empowers newcomers to the field to efficiently translate innovative ideas into successful inhaled therapies.

## Abbreviations

The following abbreviations are used in this manuscript:

API	Active Pharmaceutical Ingredient
CAGR	Compound Annual Growth Rate
CDMO	Contract Development and Manufacturing Organization
CMC	Chemistry Manufacture and Controls
CRO	Contract Research Organization
DPI	Dry Powder Inhaler
iBCS	Inhalation-based Biopharmaceutics Classification System
GMP	Good Manufacturing Practices
NOAEL	No Observed Adverse Effect Level
NOEL	No Observed Effect Level
PK	Pharmacokinetic
pMDI	Pressurized Metered Dose Inhaler
TPP	Target Product Profile
SMI	soft mist inhaler
WHO	World Health Organization
